# Cell Death Pathways as Therapeutic Targets in Rhabdomyosarcoma

**DOI:** 10.1155/2012/326210

**Published:** 2012-01-12

**Authors:** Simone Fulda

**Affiliations:** Institute for Experimental Cancer Research in Pediatrics, Goethe-University Frankfurt, Komturstraße 3a, 60528 Frankfurt, Germany

## Abstract

Resistance of rhabdomyosarcoma to current therapies remains one of the key issues in pediatric oncology. Since the success of most cytotoxic therapies in the treatment of cancer, for example, chemotherapy, depends on intact signaling pathways that mediate programmed cell death (apoptosis), defects in apoptosis programs in cancer cells may result in resistance. Evasion of apoptosis in rhabdomyosarcoma may be caused by defects in the expression or function of critical mediators of apoptosis or in aberrant expression of antiapoptotic proteins. Therefore, the identification of the molecular mechanisms that confer primary or acquired resistance to apoptosis in rhabdomyosarcoma presents a critical step for the rational development of molecular targeted drugs. This approach will likely open novel perspectives for the treatment of rhabdomyosarcoma.

## 1. Introduction

The appropriate balance between cell growth and cell death is a critical factor to maintain tissue homeostasis in multicellular organisms [1]. Thus, too little cell death as well as too much proliferation can contribute to tumor formation and progression [2, 3]. Apoptosis is the form of programmed cell death that is currently best characterized [4]. Further, defects in apoptosis programs may confer resistance to cytotoxic therapies, since the success of many anticancer therapies that are currently used in the treatment of cancer such as chemo-, radio-, or immunotherapy critically depends on intact cell death pathways [5]. This implies that a better understanding of the molecular mechanisms that control cell death pathways in cancer, for example, in rhabdomyosarcoma (RMS), will likely open novel opportunities for the development of innovative treatment strategies. In this paper, the deregulation of cell death programs in RMS will be discussed as well as possibilities to target cell death pathways for therapeutic purposes.

## 2. Rhabdomyosarcoma

RMS constitutes the most frequent form of soft tissue sarcoma in childhood and comprises two major histological subtypes that are characterized by typical genetic aberrations, that is, embryonal rhabdomyosarcoma (ERMS) and alveolar rhabdomyosarcoma (ARMS) [6]. Loss of heterozygosity (LOH) at chromosome 11p15.5 is often found in ERMS. ARMS frequently harbors reciprocal chromosomal translocations between chromosome 2 or chromosome 1 and chromosome 13, that is, t(2; 13) and t(1; 13) resulting in chimeric fusion genes composed of the DNA-binding domain of *PAX3* or *PAX7 *from chromosome 2 or 1 and the transactivation domain of *FOXO1A *from chromosome 13 [7]. Furthermore, the p53 pathways are frequently altered in RMS with p53 mutations in about half of the cases [7]. Alternatively, MDM2 amplification or overexpression may cause inactivation of the p53 pathway in RMS [7]. Although the prognosis is usually more favorable in ERMS compared to ARMS, the overall survival for advanced metastatic disease still remains poor despite aggressive treatment regimens [8, 9]. This underscores that innovative treatment strategies are required to improve patients' survival.

## 3. Apoptosis Pathway

Two key apoptosis signaling pathways can be distinguished, that is, the extrinsic (death receptor) pathway and the intrinsic (mitochondrial) pathway. Activation of either pathway typically results in the activation of caspases, a group of cysteine proteases that constitute the central execution machinery of apoptosis [4]. Activation of the extrinsic pathway following ligation of death receptors leads to the activation of caspase-8 [10]. Death receptors are members of the tumor necrosis factor (TNF) receptor gene superfamily and comprise as prominent examples TNF receptor 1 (TNFRI), CD95 (APO-1/Fas), and TNF-related apoptosis-inducing ligand (TRAIL) receptors [10]. Caspase-8 activation can result in direct activation of caspase-3. Alternatively, caspase-8 can cleave and thereby activate Bid into tBid, which translocates to mitochondria to initiate the mitochondrial pathway of apoptosis. The mitochondrial pathway involves the release of cytochrome c, apoptosis-inducing factor (AIF), or Smac/DIABLO from the mitochondrial intermembrane space into the cytosol [11]. Cytochrome c then drives the activation of caspase-3 via the apoptosome, while Smac functions in a proapoptotic manner by antagonizing “Inhibitor of Apoptosis” (IAP) proteins [11]. IAP proteins are a family of antiapoptotic proteins with eight human analogues, including XIAP, cIAP1, and cIAP2 [12]. IAP proteins block apoptosis signaling at a central node by interfering with the activation of effector caspases [12]. Another level of regulation is provided by the Bcl-2 family of proteins. They comprise both antiapoptotic proteins (i.e., Bcl-2, Bcl-X_L_, Mcl-1) as well as proapoptotic molecules (i.e., Bax, Bak, and BH3 domain only proteins) [13].

## 4. Modulation of Apoptosis Signaling Pathways for the Treatment of Sarcoma

Based on the considerable advances that were made during the last two decades in the understanding of the regulation of apoptosis pathways in human cancers, a number of strategies were developed to target apoptosis signaling pathways for therapeutic purposes. These approaches aim at shifting the balance between pro- and antiapoptotic signals towards the induction of apoptosis either by directly engaging proapoptotic molecules or pathways or indirectly by antagonizing antiapoptotic breaks. Prominent examples include the engagement of death receptors on the cell surface by ligands or antibodies, the inhibition of antiapoptotic proteins of the Bcl-2 families, and the neutralization of IAP proteins. These strategies and their implications for RMS are discussed in the following paragraphs.

## 5. Targeting Death Receptors via TRAIL in RMS

RMS cell lines were found to be relatively responsive towards the death receptor ligand TRAIL compared to their resistance to CD95-induced cell death [14]. Nevertheless, cases of resistance to TRAIL-induced apoptosis have also been described for RMS and have been attributed to different mechanisms. For example, high expression levels of antiapoptotic proteins such as cFLIP or Bcl-2 have been linked to TRAIL resistance in RMS [14, 15]. In addition, the serine/threonine kinases casein kinase I or casein kinase II have been reported to block TRAIL-induced apoptosis by interfering with the recruitment of FADD and procaspase-8 to the death-inducing signaling complex (DISC) [16, 17].

Moreover, epigenetic silencing of caspase-8 has been identified as a resistance mechanism in response to death receptor stimulation in a small proportion of RMS cell lines and primary tumor specimens [14, 18, 19]. Inactivation of caspase-8 by hypermethylation of regulatory sequences of the caspase-8 gene occurs in several human cancers in addition to RMS, for example, in neuroblastoma, medulloblastoma, Ewing tumors, and small-cell lung cancer both in cell lines as well as in primary tumor samples [20]. Of note, upregulation of caspase-8 expression restored the sensitivity to death-receptor- or anticancer drug-induced apoptosis [18, 21–25]. RMS cell lines were found to primarily express TRAIL receptor 2 (TRAIL-R2), one of the two agonistic TRAIL receptors [26]. Accordingly, a TRAIL-R2-targeted therapeutic antibody proved to be effective *in vitro* against the majority of RMS cell lines and exerted potent antitumor activity against established RMS xenografts *in vivo* [26]. Also, a strong correlation between the sensitivity to a TRAIL-R2 agonistic antibody and caspase-8 expression was reported in line with the notion that caspase-8 presents a critical regulator of the death receptor pathway of apoptosis, which is epigenetically silenced in a subset of RMS [26].

To enhance the antitumor activity of TRAIL, combination regimens were developed together with DNA-damaging agents such as chemotherapeutic drugs or *γ*-irradiation. For example, doxorubicin was reported to potentiate TRAIL-induced cytotoxicity in RMS cell lines [27, 28]. Furthermore, the proteasome inhibitor MG132 enhanced TRAIL-induced apoptosis via upregulation of TRAIL-R2 expression [29]. In a melphalan-resistant RMS cell line, TRAIL increased melphalan-induced cytotoxicity via a caspase-2- and -3-dependent mechanism [30].

In an attempt to deliver TRAIL to the tumor site to trigger apoptosis, bone-marrow-derived mesenchymal stroma cells (BM-MSCs) were efficiently transduced to express a TRAIL vector [31]. Interestingly, TRAIL-expressing MSCs were reported to effectively kill RMS cells *in vitro* [31]. Along the same lines, TRAIL expression on cytokine-induced killer (CIK) cells has been linked to CIK-mediated antitumor activity [32]. These studies indicate that cell-mediated delivery of TRAIL to RMS might present a useful strategy to engage the death receptor pathway of apoptosis in RMS.

## 6. Bcl-2 Family of Proteins

The Bcl-2 family of proteins consists of a number of both antiapoptotic as well as proapoptotic proteins [13]. It is important to keep in mind that the sensitivity to apoptosis is primarily regulated by the ratio of antiapoptotic versus proapoptotic Bcl-2 proteins rather than the expression levels of one particular Bcl-2 family protein.

Alterations of Bcl-2 family proteins frequently occur in human cancers. Genetic aberrations may contribute to aberrantly high expression of antiapoptotic Bcl-2 proteins. For example, chromosomal translocation or oncogenic activation of survival pathways may cause increased expression levels of Bcl-2 family proteins. In RMS, both PAX3 and PAX3/FKHR transcription factors were reported to transcriptionally stimulate Bcl-X_L_ mRNA expression [33] providing genetic links between RMS tumorigenesis and activation of antiapoptotic programs. Furthermore, several antiapoptotic proteins, including Bcl-2, Bcl-X_L_, and Survivin, belong to the target genes of signal transducers and activators of transcription 3 (STAT3), which has been shown to be constitutively activated in RMS cell lines [34]. Accordingly, treatment with a small molecule STAT3 inhibitor, that is, XZH-5, caused suppression of Bcl-2, Bcl-X_L_, and Survivin and resulted in increased apoptosis in RMS cells [35]. In addition, there is evidence that increased activity of the hedgehog pathway may trigger upregulation of Bcl-2 proteins. Enhanced hedgehog pathway signaling is typically associated with elevated levels and activity of Gli proteins, transcription factors that mediate the transcriptional activation of hedgehog target genes [36]. Of note, Gli proteins were reported to stimulate transcription from the Bcl-2 promoter [37]. More specifically, Gli-1 was shown to transactivate the Bcl-2 promoter, while Gli-3 inhibits transactivation by Gli-1 [37]. The notion that Bcl-2 is controlled at the transcriptional level by the hedgehog pathway is further supported by data obtained in a hedgehog-driven murine model of RMS. Accordingly, RMSs that develop in Ptch1 deficient mice were reported to display increased Bcl-2 levels [38].

An immunohistochemical study revealed that both Bcl-2 and Bax were expressed in RMS tumor samples [39]. Of note, patients whose tumors exhibited Bax expression were reported to experience a significantly longer overall survival [39]. Also, Bax expression was found to be associated with increased chemosensitivity of RMS cells to doxorubicin and actinomycin D [40]. In addition, Bax activation was described to be required for sphingosine-induced apoptosis in RMS cell lines [41]. Besides Bcl-2 and Bax, overexpression of Mcl-1, another antiapoptotic protein of the Bcl-2 family, was reported in RMS [42]. Recently, the glycolytic inhibitor 2-deoxyglucose (2-DG) was reported to trigger apoptosis in alveolar, but not embryonal, RMS cell lines [43]. Induction of cell death was accompanied by downregulation of Mcl-1 as well as upregulation of Noxa, a proapoptotic Bcl-2 family protein [43].

Since high expression levels of antiapoptotic Bcl-2 proteins can confer resistance to current treatment approaches by blocking the activation of the mitochondrial pathway, several strategies have been developed in recent years to antagonize the cytoprotective functions of antiapoptotic Bcl-2 proteins. A prominent example is small molecule Bcl-2 inhibitors, which interfere with the protein-protein interaction site between antiapoptotic Bcl-2 proteins and the multidomain proteins Bax or Bak [44]. Despite overexpression of antiapoptotic Bcl-2 proteins in RMS, ABT-263, a second-generation compound that inhibits Bcl-2, Bcl-X_L_, and Bcl-w, demonstrated limited *in vivo* activity against the PPTP's solid tumor panels including RMS when tested as single agent [45]. This indicates that rational combination treatments may be required to exploit Bcl-2 inhibitors in RMS.

## 7. IAP Proteins

Sensitivity or resistance to apoptosis may also be controlled by IAP proteins, for example, XIAP, cIAP1, and cIAP2 [12]. Among the IAP proteins, XIAP exhibits the strongest antiapoptotic properties and blocks apoptosis by binding to and inhibiting active caspase-3 and -7 and by interfering with the activation of caspase-9 [12]. IAP proteins are considered as promising targets for therapeutic intervention in human cancers. However, little is yet known about the role and relevance of these proteins in RMS. cIAP2 was shown to be upregulated following exposure to hypoxia in RMS cells and may contribute to apoptosis resistance under these conditions [46]. Notably, downregulation of XIAP by the antisense oligonucleotide AEG35156 was recently reported to induce apoptosis in RMS cells [47]. These findings point to a potential impact of strategies that antagonize IAP proteins in RMS and suggest that additional studies are required to evaluate IAP proteins as therapeutic targets in RMS. 

## 8. Conclusions

Programmed cell death is a key regulator of tissue homeostasis and plays a central role in tumorigenesis. In addition, intactness of apoptosis pathways constitutes a critical determinant of the sensitivity versus resistance of RMS to most current treatment strategies. The discoveries over the last years on the regulation of apoptosis signaling pathways in RMS resulted in the identification of new molecular targets, which can be exploited for the development of innovative experimental therapies, for example, TRAIL receptor agonists, small molecule inhibitors of antiapoptotic Bcl-2 proteins, or strategies targeting IAP proteins. One key challenge in the future will be to develop biomarkers in order to identify the group(s) of patients that will profit the most from these molecular targeted therapies. Also, it will likely be critical to develop rational combination therapies using these signal transduction modulators together with conventional cancer therapies such as chemo- or radiotherapy in order to exploit synergistic interactions. Hopefully, this strategy to engage cell death signaling pathways in RMS cells will open new avenues for the treatment of RMS patients.
